# Recollecting Cross-Cultural Evidences: Are Decision Makers Really Foresighted in Iowa Gambling Task?

**DOI:** 10.3389/fpsyg.2020.537219

**Published:** 2020-12-21

**Authors:** We-Kang Lee, Ching-Jen Lin, Li-Hua Liu, Ching-Hung Lin, Yao-Chu Chiu

**Affiliations:** ^1^Department of Psychology, Soochow University, Taipei, Taiwan; ^2^Shin Kong Wu Ho-Su Memorial Hospital Sleep Center, Taipei, Taiwan; ^3^Department of Psychology, Kaohsiung Medical University, Kaohsiung, Taiwan; ^4^Research Center for Nonlinear Analysis and Optimization, Kaohsiung Medical University, Kaohsiung, Taiwan

**Keywords:** iowa gambling task, IGT global map, foresight, prominent deck B phenomenon, gain-loss frequency, gain-stay loss-randomize decision strategy, cross-cultural, dynamic decision-making

## Abstract

The Iowa Gambling Task (IGT) has become a remarkable experimental paradigm of dynamic emotion decision making. In recent years, research has emphasized the “prominent deck B (PDB) phenomenon” among normal (control group) participants, in which they favor “bad” deck B with its high-frequency gain structure—a finding that is incongruent with the original IGT hypothesis concerning foresightedness. Some studies have attributed such performance inconsistencies to cultural differences. In the present review, 86 studies featuring data on individual deck selections were drawn from an initial sample of 958 IGT-related studies published from 1994 to 2017 for further investigation. The PDB phenomenon was found in 67.44% of the studies (58 of 86), and most participants were recorded as having adopted the “gain-stay loss-randomize” strategy to cope with uncertainty. Notably, participants in our sample of studies originated from 16 areas across North America, South America, Europe, Oceania, and Asia, and the findings suggest that the PDB phenomenon may be cross-cultural.

## Introduction

In recent decades, the Iowa Gambling Task (IGT; Bechara et al., 1994) has gradually become a classic experimental paradigm of dynamic decision making (Dunn et al., 2006) and has even been used to clinically assess patients with ventromedial prefrontal cortex (vmPFC) dysfunction related to brain lesions (Bechara, 2007, 2016). The IGT is a dynamic task that simulates the uncertain conditions of a real-life situation. In the task, four decks are displayed with a pseudorandomized and symmetrical gain-loss schedule that is not disclosed to the participants. Based on the schedule developed by Bechara et al. (1994), decks A and B are defined as “bad decks” due to their long-term disadvantageous outcome despite a large gain (e.g., $100) in each selection, while decks C and D are scheduled with a small gain (e.g., $50) in each selection and defined as “good decks” due to their long-term advantageous outcome. Furthermore, decks A and C contain five times as many losses, while decks B and D contain an average of only one loss for every 10 trials. Compared to patients with vmPFC lesions, Bechara et al. (1994) theorized that control participants would form a “somatic marker” (Damasio, 1994) when making deck selections and that the gut feeling related to the somatic marker would lead to foresighted and rational decision making—that is, choosing “good decks” (C and D) in the IGT. Moreover, a series of studies by Bechara et al. (1994; 1997; 1998; 1999; 2000) replicated these results.

However, Dunn et al. (2006) undertook a review of IGT-related studies and noted several possible issues, including the possibility that the inconsistencies identified between prior studies’ findings were due to variability of the normal (control) participants. Recently, though, others have shown that the IGT participants in control groups typically favor bad deck B not only more than deck A, but also more than good decks C or D (Wilder et al., 1998; Toplak et al., 2005; Fernie and Tunney, 2006; Lin et al., 2007, 2013; Steingroever et al., 2013), which is inconsistent with the basic assumption proposed by Bechara et al. (1994). This finding has been defined as the “prominent deck B (PDB) phenomenon” (Lin et al., 2007), and researchers have inferred that the selection preference is due to a “gain-loss frequency effect”—that is, like good deck D, bad deck B features nine gains and one loss across 10 trials, in terms of net value (Lin et al., 2007; Chiu et al., 2008). The PDB phenomenon has been acknowledged as a critical issue in IGT-related research (Chiu et al., 2018), yet, few studies (Chiu et al., 2012; Steingroever et al., 2013) have fully examined whether it exists in relation to prior IGT-related findings.

Some researchers have attributed a preference for a particular IGT deck with high-frequency gain to cultural differences (Ekhtiari et al., 2009; Bakos et al., 2010). For example, Bakos et al. (2010) found that culture or birth country could partially influence participants’ behavior in the IGT. However, a similar finding regarding high-frequency gain preference in the IGT was also observed in a sample of Iranian participants. Ekhtiari et al. (2009) attributed the demonstration of the phenomenon in this example to the restriction on gambling within Islamic culture and the country’s relatively late development of a bourgeois class.

Chiu et al. (2012) undertook a review of the PDB phenomenon and found that out of 16 studies, 13 (81.25%) obtained results for individual deck selections (i.e., the mean selection number with respect to each deck was presented in the study) that demonstrated the PDB phenomenon. Steingroever et al. (2013) published the results of two reviews related to the IGT: the first examined 17 studies that utilized data regarding selections from four decks (479 normal participants in total); the second review examined 39 groups and the corresponding mean selections from good and bad decks (1,427 normal participants in total). The research team then sent emails requesting the raw data. After receiving responses from seven authors, the researchers collected data from 162 normal participants and analyzed the 8 data sets. Ultimately, both reviews concluded that the normal participants had a preference for low-frequency loss deck B, and the selections persisted until the end of the IGT (Steingroever et al., 2013). The issue of cultural difference, however, was not clearly specified in these review studies.

Following the findings of Chiu et al. (2012) and Steingroever et al. (2013), but in contrast to the observations made by Ekhtiari et al. (2009) and Bakos et al. (2010), we hypothesized that the PDB phenomenon (i.e., a preference of normal participants for the high-frequency gain bad deck B in the IGT) exists cross-culturally. That is, cultural difference may not be a critical factor for interpreting decision-making behavior in the IGT. To test this hypothesis, we reviewed past studies that were identified through a PubMed search of the MEDLINE biomedical database and further integrated the findings of review studies (Chiu et al., 2012; Steingroever et al., 2013) to examine the geographical distribution of IGT-related studies that found individual deck selections in the IGT and plot a global map of the PDB phenomenon.

## Methods

### Procedure

A search for IGT-related studies dating from 1994 to March 31, 2017 was performed on the MEDLINE biomedical database using the PubMed search engine and the keywords “Iowa gambling” and “Bechara card task.” We found 945 articles that featured “Iowa gambling” and 18 articles using “Bechara card task” as keywords. Once we had excluded 12 overlapping IGT-related studies, 951 IGT-related studies were individually reviewed.

### Inclusion and Exclusion Criteria

We ultimately identified 140 articles that presented deck decisions in the main text, tables, or figures. Regarding the version of the IGT, testing procedure consistencies, and the ages of participants, we excluded 22 studies that used revised versions of the IGT (e.g., the Hungry Donkey task, the inverted IGT, the simple IGT, the net-value IGT, and the Soochow Gambling Task), 9 studies that manipulated testing procedures, 9 studies that did not present the control group data, 9 studies that included participants younger than 17 years of age, 7 studies that presented the results of fewer than 100 trials, 3 studies that only presented representative data, and 2 studies in which the data for deck selection were unclear even though each selection of every participant was presented. Consequently, 79 studies that used the original IGT’s gain-loss structure and presented data for individual deck choices were further analyzed.

Table 1 presents the deck selection data of control participants in 100 IGT trials (namely, over 100 IGT trials were not depicted here) that we extracted from these 79 studies. For studies that presented figures without precise means and standard deviations, we measured and estimated the values based on the scale of the figures.

**TABLE 1:** Data of normal participants in Iowa Gambling Task (IGT)-related studies from PubMed search, which showed individual deck selections.

Authors	Sample size (sex)	Mean_age_ (SD)	Source of study	Mean number of card selection				Note
				Deck A	Deck B	Deck C	Deck D	
Petry et al., 1998	59 (26F, 33M)	35 (10)	US	16.80	26.20	28.50	32.20	≈
Petry, 2001	21 (21M)	36.1 (11.5)	US	15.50	24.80	27.80	32.70	≈
North and O’Carroll, 2001	20 (4F, 16M)	30.8 (1.91)	GB	9.80	19.90	36.60	34.90	≈
O’Carroll and Papps, 2003	11 (5F, 6M)	20.0 (3.1)	GB	15.00	26.70	20.80	35.60	≈Placebo
Overman, 2004	101 (54F, 47M)	F: 21.1, M: 19.1	US	13.00	29.25	25.50	32.25	% ≈ +Adult Female, Weather Task First Reavis and Overman, 2011
				13.00	29.70	22.10	35.25	% ≈ +Adult Female, Card Task First Reavis and Overman, 2011
				11.65	21.30	29.10	38.00	% ≈ +Adult Male, Weather Task First Reavis and Overman, 2011
				13.90	25.80	32.10	28.70	% ≈ +Adult Male, Card Task First Reavis and Overman, 2011
Shurman et al., 2005	10 (5F, 5M)	32.1 (4.5)	US	15.70 (4.1)	18.50 (6.4)	34.00 (9.0)	31.80 (6.3)	
Bark et al., 2005	26 (14F, 12M)	29.81 (9.39)	DE	22.30	29.10	24.30	24.00	≈ +
Rodriguez-Sanchez et al., 2005	22 (10F, 12M)	26.09 (6.49)	ES	16.91 (5.51)	32.05 (13.22)	20.50 (8.55)	30.55 (14.04)	
Fernie and Tunney, 2006	20 (not reported)	Not reported	GB	18.80 (1.45)	31.60 (2.25)	21.05 (1.73)	28.55 (2.22)	Session 1 Hint-Fascimile group
	20 (not reported)	Not reported	GB	20.45 (1.31)	32.65 (2.02)	21.65 (1.44)	25.25 (1.65)	Session 1 No Hint-Fascimile group
Northoff et al., 2006	14 (7F, 7M)	28.7 (19–34)	DE	15.19	26.61	18.78	38.84	= +
Kester et al., 2006	25 (11F, 14 M)	17.1 (1.8)	US	20.70 (5.1)	25.20 (6.5)	25.60 (10.3)	28.40 (10.3)	
Sevy et al., 2007	20 (8F, 12M)	33 (10)	US	18.00 (5)	31.00 (8)	23.00 (6)	27.00 (6)	
Lee et al., 2007	28 (13F, 15M)	26.9 (3.6)	KR	17.60 (6.2)	23.60 (7.7)	25.50 (10.9)	33.10 (13.5)	
Martino et al., 2007	15 (9F, 6M)	34.96 (10.93)	AR	15.20 (3.74)	26.66 (10.46)	21.13 (9.25)	37.00 (8.75)	
Zamarian et al., 2008	33 (22F, 11M)	36.1 (13.7)	AT	12.50 (4.2)	21.40 (8.0)	25.60 (10.8)	40.40 (11.9)	Young adults
	52 (34F, 18M)	69.3 (7.0)		15.20 (5.4)	26.60 (9.0)	22.50 (8.4)	35.70 (9.5)	Old adults
Ahn et al., 2008	36 (18F, 18M)	22.0 (18–33)	US	13.28	24.30	31.60	30.82	% ≈ +
Viswanath et al., 2009	25 (10F, 15M)	27.44 (6.40)	IN	20.68 (7.23)	22.84 (7.24)	26.04 (6.54)	31.24 (8.40)	
van den Bos et al., 2009	10 (10M)	24.7 (0.5)	NL	16.80 (2.5)	24.00 (2.8)	27.80 (3.0)	31.40 (2.6)	Male control subjects
	12 (12F)	22.3 (0.4)	NL	21.30 (1.9)	25.20 (1.5)	26.40 (1.9)	27.10 (1.1)	Female control subjects
Kim et al., 2009	55 (26F, 29M)	28.8 (7.5)	KR	16.90	26.95	24.30	33.90	≈
van Toor et al., 2011	31 (15F, 16M)	36.32 (12.36)	NL	15.26 (6.78)	24.23 (9.29)	21.97 (7.87)	38.55 (15.70)	
Martino et al., 2011	34 (22F, 12M)	40.0 (12.9)	AR	14.70 (6.3)	27.10 (12.1)	20.40 (10.9)	37.80 (12.5)	
Adida et al., 2011	150 (75F, 75M)	38.8 (10.6)	GB FR	17.10	24.70	25.70	32.80	≈
Kim et al., 2011	21 (21M)	30.52 (2.98)	KR	18.05 (8.29)	20.19 (7.52)	29.67 (12.13)	32.10 (12.93)	
Tchanturia et al., 2012	61 (41F, 20M)	22.2 (5.68)	GB ES	17.29 (6.94)	25.37 (9.04)	25.39 (10.77)	31.95 (11.57)	Female
		25.45 (7.63)		12.45 (6.71)	27.15 (14.85)	29.75 (12.54)	30.65 (15.21)	Male
Gansler et al., 2011	214 (123F, 91M)	54.65 (17.44)	US	15.52 (6.23)	28.69 (11.68)	20.87 (8.73)	34.92 (14.68)	
Visagan et al., 2012	30 (16F, 14M)	22.2 (3.7)	GB	11.00	28.00	23.00	33.10	≈ +
Mogedas Valladares and Alameda-Bailen, 2011	33 (18F, 15M)	29.91 (9.45)	ES	14.48 (5.149)	26.94 (7.905)	29.30 (6.502)	29.27 (5.986)	
Escartin et al., 2012	31 (14F, 17M)	50 (11.1)	ES	17.97 (6.98)	26.45 (12.05)	25.39 (13.31)	30.13 (11.15)	
Gansler et al., 2012	124 (65F, 59M)	54.9 (16.4)	US	15.94 (6.62)	29.18 (11.20)	20.46 (7.99)	34.43 (13.99)	Same subjects as Gansler et al., 2011but exclude Connecticut subjects.
Upton et al., 2012	27 (5F, 22M)	35 (10.44)	AU	16.64	34.88	17.76	31.26	% ≈ +
Gescheidt et al., 2012	20 (5F, 15M)	49.95 (9.03)	CZ	20.50	24.40	25.30	29.80	≈ +
Horstmann et al., 2012	119 (66F, 53M)	F: 25.2 (4.9) M: 24.7 (3.1)	DE	13.92	33.59	21.71	30.79	= +
Alameda-Bailen et al., 2012	41 (14F, 27M)	25.17 (5.652)	ES	15.95 (5.731)	24.49 (6.874)	29.61 (7.334)	29.95 (6.618)	
Carvalho et al., 2012	40 (22F, 18M)	25.50 (4.70)	BR	14.75 (6.27)	30.90 (13.43)	20.88 (10.06)	34.28 (11.18)	Young adults
	40 (30F, 10M)	67.40 (5.02)		18.90 (6.77)	29.23 (11.14)	22.30 (6.72)	29.85 (11.52)	Elderly adults
Steingroever et al., 2013	162 (82F, 80M)	25.56 (4.86)	NL	15.00	35.00	20.00	30.00	
Worthy et al., 2013a	41 (30F, 11M)	21.29 (18–29)	US	14.78	34.24	25.36	25.72	% ≈ +
van den Bos et al., 2013	213 (140F, 73M)	Not reported	NL	18.70	26.90	27.00	27.20	≈Female (the reconstruct data)
				18.60	25.20	27.80	28.20	≈Male (the reconstruct data)
Miller et al., 2013	77 (77F)	17–25	US	14.10 (5.02)	31.84 (14.02)	20.99 (11.61)	33.06 (13.85)	
Le Berre et al., 2014	45 (7F, 38M)	44.76 (7.78)	FR	20.31 (7.87)	20.22 (6.07)	30.69 (6.86)	28.78 (7.18)	
Kim et al., 2012	33 (17F, 16M)	27.8 (3.0)	KR	17.10 (7.1)	24.40 (9.7)	27.50 (13.8)	30.80 (14.0)	
Penolazzi et al., 2013	84 (44F, 40M)	26.47 (7.14)	IT	14.60	30.80	22.30	33.90	≈
Lin et al., 2013	72 (37F, 35M)	Not reported	TW	18.69	30.53	22.99	27.79	
Vassileva et al., 2013	12 (12F)	33.5 (8.5)	US	16.90	33.10	22.50	27.70	≈HIV-seronegative/no crack cocaine and/or heroin use history
Kloeters et al., 2013	28 (12F, 16M)	64.2 (4.4)	AU	12.30 (4.5)	28.20 (12.9)	20.30 (10.1)	39.20 (13.6)	
Buelow and Suhr, 2014	70 (48F, 22M)	18.94 (1.21)	US	18.16	29.22	22.19	30.44	% +
Lavin et al., 2014	10 (5F, 5M)	23.4 (2.4)	CL	21.30	34.80	20.00	23.90	≈ +
Beitz et al., 2014	17–29 y/o: 664 (485F, 179M) 30–59 y/o: 281 (211F, 70M)	17–89	US	14.90	25.30	25.80	35.10	≈ +17–59 y/o group
	60–89 y/o: 293 (202F, 91M)			15.40	29.00	21.80	34.90	≈ +60–89 y/o group
Cotrena et al., 2014	55 (27F, 28M)	33.4 (17.4)	BR	17.00	25.80	23.00	36.50	≈
Wolk et al., 2014	17 (8F, 9M)	36.53 (12.10)	DE	16.44 (10.10)	35.25 (11.70)	20.31 (11.60)	28.00 (14.50)	
Cardoso et al., 2014	18 (14F, 4M)	59.28 (10.25)	BR	16.00 (6.48)	22.61 (7.49)	25.61 (6.58)	36.65 (12.36)	
LeGris et al., 2014	41 (41F)	31.2 (9.0)	CA	13.40 (5.2)	26.05 (12.3)	23.34 (13.4)	37.20 (14.5)	
Lee et al., 2014	52 (26F, 26M)	21.39 (3.64)	TW	15.28 (5.75)	31.98 (8.32)	25.54 (11.32)	27.20 (9.25)	
Alameda-Bailen et al., 2014	63 (26F, 37M)	25.11 (6.01)	ES	15.60	23.80	30.50	30.50	≈+
Hong et al., 2015	30 (6F, 24M)	29.1 (7.6)	CN	19.70	21.60	31.40	29.00	≈
Seeley et al., 2014	92 (76F, 16M)	Not reported	CA	18.15	33.10	21.10	29.05	% ≈ +Session 1
Matsuzawa et al., 2015	50 (17F, 33M)	31.9 (7.8)	JP	20.90	29.10	26.10	24.40	≈
Evans and Hampson, 2015	93 (48F, 45M)	19.69 (17–28) 19.54 (17–32)	CA	15.60 17.00	31.10 34.80	21.80 23.30	34.20 27.10	≈ +Male ≈ +Female
Hori et al., 2014	51 (26F, 25M)	36.7 (9.9)	JP	17.70 (6.9)	26.00 (12.2)	30.70 (12.6)	25.20 (8.9)	
Ma et al., 2015	24 (24M)	21.7 (1.8)	CN	19.70	31.50	24.70	24.10	≈ +
Bull et al., 2015	50 (30F, 20M)	21.44 (3.79)	NZ	16.10	21.30	30.80	31.80	≈
Brown et al., 2015	43 (17F, 26M)	41.1 (11.8)	US	15.00	29.40	24.60	31.70	≈
Zhang et al., 2015c	88 (41F, 47M)	19.17 (1.29)	CN	25.00	30.40	21.40	23.90	≈Low trait anxiety group
	119 (57F, 62M)	19.17 (1.29)	CN	23.90	24.60	21.80	30.00	≈Medium trait anxiety group
	97 (45F, 52M)	19.17 (1.29)	CN	30.00	23.10	19.60	27.70	≈High trait anxiety group
Smart and Krawitz, 2015	25 (10F, 15M)	69.88 (3.36)	CA	11.77	27.11	17.86	41.39	% ≈ +
Besnard et al., 2015	17 (2F, 15M)	44.1 (9–76)	FR	12.40 (7.9)	19.60 (5.3)	29.90 (6.4)	38.00 (10.1)	
Huang et al., 2015	65 (42F, 23M)	24.50 (3.79)	US	15.80	34.10	17.80 (10.28)	31.20 (14.74)	≈Younger adult
	65 (47F, 18M)	75.28 (6.40)		16.70	34.40	21.49 (9.72)	26.86 (11.64)	≈Older adult
Zhang et al., 2015b	80 (13F, 67M)	19.2 (2.96)	CN	24.30	24.30	22.70	29.00	≈
Alarcon et al., 2015	40 (Not reported)	Not reported	ES	18.60	32.90	19.80	28.70	≈ +Original IGT group
Zhang et al., 2015a	115 (55F, 60M)	27.32 (7.81)	CN	24.40	23.70	22.80	29.10	≈
Seeley et al., 2016	13 (Not reported)	Not reported	CA	20.36	36.10	16.81	26.46	% ≈ +Session 1
Okdie et al., 2016	30 (Not reported)	Not reported	US	16.90	31.14	22.60	28.26	% +Study 1: control group
	30 (Not reported)	18.18 (0.48)		15.96	33.70	20.04	30.30	% +Study 2: control group
Piper et al., 2016	47 (28F, 19M)	18.8 (0.3)	US	16.20 16.80	32.80 33.00	24.60 21.50	25.90 29.00	≈PAR version ≈PEBL version
Besnard et al., 2016	30 (22F, 8M)	55.1 (22.6)	FR	16.00 (6.2)	21.30 (6.8)	29.00 (8.4)	33.70 (6.2)	
Hawthorne and Pierce, 2015	30 (Not reported)	18–29	US	13.90	28.90	29.60	28.10	≈Full attention group
Pedersen et al., 2017	38 (16F, 22M)	40 (13.8)	DE	19.10 (6.4)	30.70 (10.1)	19.60 (11.3)	30.60 (10.0)	
Lin et al., 2016	145 (43F, 102M)	18.6 (0.97)	TW	17.39	32.47	24.73	25.41	
Yechiam et al., 2016	130 (65F, 65M)	23.5 (18–28)	IL	12.50	26.30	26.30	34.40	Study 2
Visser-Keizer et al., 2016	59 (22F, 37M)	43.50 (1.90)	NL	14.90 (7.7)	29.30 (14.1)	25.20 (19.6)	30.60 (18.1)	
Wright et al., 2017	36 (Not reported)	Not reported	GB	17.50	23.80	25.60	30.90	≈ +
Jollans et al., 2017	20 (9F, 11M)	24.9 (4.8)	GB	18.82	34.49	21.10	25.96	% ≈ +

*Note: AR, Argentina; AT, Austria; AU, Australia; BR, Brazil; CA, Canada; CL, Chile; CN, China; CZ, Czechia; DE, Germany; ES, Spain; FR, France; GB, United Kingdom; IL, Israel; IN, India; IT, Italy; JP, Japan; KR, South Korea; NL, Netherlands; NZ, New Zealand; TW, Taiwan; and US, United States of America. The abbreviation codes were based on the “ISO 3166-1 alpha-2” (https://en.wikipedia.org/wiki/List_of_ISO_3166_country_codes). Note: The first step of data redrawn from the figure is to measure the length (mm) of a single unit of the *Y*-axis. Accordingly, the second step is to convert each data point in the figure to how many card selections. Notably, there are some potential small errors (e.g., the length of the first unit is not totally equal to that of the second unit of *Y*-axis in the same figure) in the procedure of estimation, so we have to make a note here; some of the estimated data sets were summed into 100 and some are not. ≈Data were transcribed from figures. ≈ +Data of average deck selection in blocks were transcribed and summed. = +Data of average deck selection in blocks were summed. % +Data of average deck selection percentage in blocks were calculated and summed. % ≈ +Data of average deck selection percentage in blocks were transcribed, calculated, and summed. Note without special signs above: numerical deck selection data were obtained from original studies.*

To increase the integrity of reviewing IGT-related studies, we investigated the studies originally reviewed by Chiu et al. (2012)and Steingroever et al. (2013)that had focused on the issue of high-frequency gain deck preference in the IGT (see Table 2). Six studies were selected after we excluded repeated articles from the database mentioned above. The original selection data of 100 trials in a concurrent IGT condition published in Chiu et al. (2012)were also obtained and included in the present research. In total, there were seven studies sourced from Chiu et al. (2012)and Steingroever et al. (2013).

**TABLE 2:** Data of normal participants in IGT-related studies included in Chiu et al. (2012)and Steingroever et al. (2013).

Authors	Sample size (sex)	Mean_age_(SD)	Source of study	Mean number of card selection				Note
				Deck A	Deck B	Deck C	Deck D	
Bechara et al., 1994	44 (21F, 23M)	Not reported	US	14.00	16.00	35.00	35.00	≈
Wilder et al., 1998	30 (18F, 12M)	30.2 (9.7)	US	20.20 (5.8)	26.80 (7.0)	24.10 (7.9)	28.90 (7.6)	
Tomb et al., 2002	10 (5F, 5M)	Not reported	US	15.00	19.00	34.00	32.00	≈
Ritter et al., 2004	15 (15M)	47.1 (10.2)	US	18.00	25.00	24.00	33.00	≈
Caroselli et al., 2006	141 (73F, 68M)	21.7 (4.6)	US	22.00	35.00	20.00	23.00	≈
Fum et al., 2008	Not reported	Not reported	IT	14.87	32.84	17.09	35.19	Experiment 1 standard condition
Chiu et al., 2012	24 (12F, 12M)	Not reported	TW	18.13	31.50	25.71	24.67	100 trials selection data obtained from authors

*Note. IT, Italy; TW, Taiwan; and US, United States of America. ≈Data were transcribed from tables of the review studies.*

### Study Selection and Data Extraction

Two authors (W-KL and C-JL) independently retrieved the studies that presented individual deck selections (i.e., in the main text, tables, or figures). They independently reviewed each study to extract the data and measured the average selection numbers (i.e., based on the scale of the figures). Any disagreement with respect to the process of study selection or data extraction was resolved through consensus via repeated measurements and discussion. All average numbers of choice obtained through measurement by two researchers were controlled under the difference ≤1 selection approach.

### Data Analysis

Data analysis was performed on a total of 86 studies, 79 of which were retrieved from the MEDLINE biomedical database and 7 from the 2 review studies noted above (Chiu et al., 2012; Steingroever et al., 2013). Notably, each experimental condition performed by normal participants in the 86 studies was considered as a single data set, even though there may in fact have been 2 (Fernie and Tunney, 2006; Zamarian et al., 2008; van den Bos et al., 2009, 2013; Carvalho et al., 2012; Tchanturia et al., 2012; Beitz et al., 2014; Evans and Hampson, 2015; Huang et al., 2015; Okdie et al., 2016; Piper et al., 2016), 3 (Zhang et al., 2015c), or 4 experimental conditions (Overman, 2004) in the original study (experimental conditions are marked in the note of Table 1). In total, 102 data sets obtained from 86 studies were subsequently analyzed.

**FIGURE 1:**
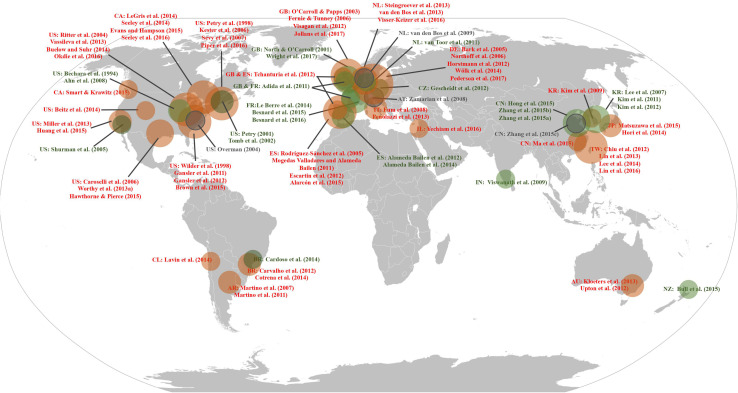
The Iowa Gambling Task (IGT) global map. The figure illustrates the geographical distribution of IGT-related studies that showed individual deck selections. Red circles indicate studies demonstrating the PDB phenomenon, green circles indicate studies that support the original IGT assumptions, and gray circles indicate studies that were unclassifiable. AR, Argentina; AT, Austria; AU, Australia; BR, Brazil; CA, Canada; CL, Chile; CN, China; CZ, Czechia; DE, Germany; ES, Spain; FR, France; GB, United Kingdom; IN, India; IT, Italy; JP, Japan; KR, South Korea; NL, Netherlands; NZ, New Zealand; TW, Taiwan; and US, United States of America. Adapted from “Robinson projection, national borders, areas grouped” (https://en.wikipedia.org/wiki/Wikipedia:Blank_maps#/media/File:BlankMap-World.svg) in the public domain.

To verify whether a “gain-stay loss-randomize” decision strategy was demonstrated in the different data sources, we conducted a decks-by-groups repeated measures analysis of variance (ANOVA) using IBM SPSS Statistics (version 22) and analyzed individual deck selection data. To visualize whether the PDB phenomenon is cross-cultural, all studies that presented each of four deck selections obtained from the database search and review studies were marked on an IGT global map according to the source and origin of the study’s participants (Figure 1). We defined selections of bad deck B equal to 25 or more (i.e., higher than the randomized choices of 100 trials, or chance level), as being a “PDB phenomenon.”

This standard was strictly applied while we were identifying whether the PDB phenomenon existed in the 86 studies—specifically, every experimental condition performed by normal participants had to consistently exhibit the PDB phenomenon, even in studies that featured more than one experimental condition (as discussed above). As a result, there were four studies (Overman, 2004; Zamarian et al., 2008; van den Bos et al., 2009; Zhang et al., 2015c) that we were unable to classify due to the phenomenon existing inconsistently across different experimental conditions: the PDB phenomenon existed in only three of the four experimental conditions in Overman (2004), one of the three experimental conditions in Zhang et al. (2015c), and one of the two experimental conditions in Zamarian et al. (2008)and van den Bos et al.(2009; see Table 1and Figure 1). Although unclassifiable in the global map (see gray circles in Figure 1), the data sets from the four studies were still included in the following analysis.

## Results

The repeated measures ANOVA showed that the interaction effect of groups (studies retrieved from the database search and review studies) and decks was nonsignificant, *F*(2.377, 237.73) = 0.445, *p*= 0.676 (Greenhouse–Geisser correction). The main effect of the decks was significant, *F*(2.377, 237.73) = 39.141, *p*< 0.001, *η^2^*= 0.281, but that of the groups was not, *F*(1, 100) = 0.123, *p*= 0.726. In short, the results indicated no difference between the data obtained from the MEDLINE database and the review studies (Chiu et al., 2012; Steingroever et al., 2013).

**FIGURE 2:**
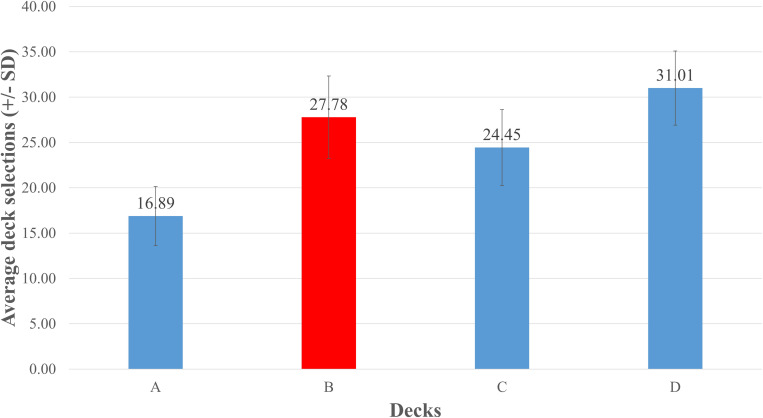
Mean number of card selections in 86 IGT-related studies. The figure was produced by averaging the numbers of the four decks chosen across the 86 IGT-related studies. Selections of deck B were relatively higher than those of decks A and C, demonstrating that the PDB phenomenon was present. This finding is consistent with those obtained in a growing number of other IGT-related researches.

As there was no difference between the two data sources, we combined the data obtained from the two sources (86 studies in total) and further conducted a repeated measures ANOVA to test for differences between decks. The results showed a significant difference with respect to the selections of individual decks, *F*(2.379, 240.293) = 171.702, *p*< 0.001, and *η^2^*= 0.63 (Greenhouse–Geisser correction). The selection of deck B was significantly higher than that of decks A, *p*< 0.001, and C, *p*< 0.001. Moreover, the selection of deck C was higher than that of deck A, and the selection of deck D was higher than those of all other decks, *p*s < 0.001. These results suggest that the PDB phenomenon was common in the reviewed studies (Figure 2).

Combining the results of the studies from the MEDLINE database and the review studies, we found that 67.44% (58 of 86) featured a selection of the disadvantageous deck B ≥ 25 times, and this preference corresponded to our definition of the PDB phenomenon (detailed above). As shown in Figure 1, the normal participants in these 58 studies originated from 16 regions of North America, South America, Europe, Oceania, and Asia: specifically, Argentina, Australia, Brazil, Canada, Chile, China, Germany, Israel, Italy, Japan, Netherlands, South Korea, Spain, Taiwan, United Kingdom, and the United States.

## Discussion

Most IGT-related studies have used the calculation (C + D) − (A + B) to define decision-making performance. Correspondingly, the basic assumption of the IGT (Bechara et al., 1994, 1997) posited that normal (control group) participants could perform advantageously and make rational decisions guided by implicit emotion, in contrast to participants who were unable to access an emotional system due to a vmPFC lesion. However, the present article found that in 67.44% (58 out of 86) of the IGT-related studies that showed individual deck selection data, a preference for the disadvantageous deck B was observed. The participants in these 58 studies originated from 16 different areas across North America, South America, Europe, Asia, and Oceania. Therefore, we infer that the PDB phenomenon in the IGT is cross-cultural.

### Individual and Cultural Issues in the IGT

A prior critical review article (Dunn et al., 2006) reiterated Bechara and Damasio’s (2002)finding that about 20% of normal participants performed poorly in the IGT. In fact, Bechara and Damasio (2002)showed that 37% of normal (control group) participants performed within the range of vmPFC patients, referring to the criterion of the net score (C + D) − (A + B) < 10. The present research showed that in more than 60% of sample studies, normal participants consistently favored the disadvantageous deck B. This PDB phenomenon is evidently different from the results obtained by Bechara and Damasio (2002).

Previous IGT-related studies have attributed participants’ preference for decks with a high-frequency gain (decks B and D) to cultural issues. For example, Bakos et al. (2010)postulated that a preference for deck B only existed in certain cultures and further investigated decision-making differences between Brazilians and Americans. In their study, 17% of the Brazilian participants in an IGT were categorized as “normal decision makers,” compared to the 60% of American participants who were categorized as normal, according to the measure (C + D) − (A + B) > 18, a criterion proposed by Denburg et al. (2005). These results suggested that Americans perform better than Brazilians in the IGT; Bakos et al. (2010)posited that the difference might relate to capitalism in the United States making the daily lives of Americans much more reliant on their ability to manage financial issues compared to Brazilians. However, the study did not clarify whether a preference for decks with a high-frequency gain existed in both Americans and Brazilians.

Similar to our study, Ekhtiari et al. (2009)performed an analysis of individual decks and found that Iranian participants favored the high-frequency gain decks B and D. The researchers attributed the phenomenon to two possible causes: (1) the limitations on gambling under Islamic law meant that Iranian participants were unclear about or lacking gambling concepts, which further affected their decision-making performance in the IGT according to frequency-based valuations; and (2) the late development of a bourgeois class in Iran, and therefore of concepts such as land ownership and work ownership, meant that the country’s workers lacked long-term decision-making experience (Ekhtiari et al., 2009). In contrast to the cultural difference perspective, we suggested a cross-cultural preference for high-frequency gain decks B and D in the IGT. This is supported by our confirmation that the phenomenon exists in 16 areas across North America, South America, Europe, Asia, and Oceania.

However, although our sample of studies showed that the PDB phenomenon existed cross-culturally, the finding was limited by the lack of analysis regarding cultural factors. Future studies could further analyze the performance of normal participants under different cultural factors (e.g., Western or Eastern cultural contexts) and examine whether the PDB phenomenon exists universally.

### Methodological Issue in Iowa Gambling Task-Related Studies

Furthermore, of the 951 IGT-related studies originally sourced through the MEDLINE database, only 140 showed individual deck data, and most of the remaining 811 studies used the calculation (C + D) − (A + B) to differentiate the performances of clinical versus control participants. It is possible that the scoring method may have obscured the existence of the PDB phenomenon in control participants (Chiu and Lin, 2007; Lin et al., 2007, 2013; Steingroever et al., 2013) and further neglect differences between the preferences of clinical versus control participants for individual decks. Consequently, researchers may be missing opportunities to observe differences regarding more specific decision-making patterns.

In the present research, we further analyzed the 86 studies that featured individual deck data according to the criterion (C + D) − (A + B) < 10, as used by Bechara and Damasio (2002). According to this analysis, 45.35% (39 out of 86) of the studies showed that normal participants performed within the range of vmPFC patients (see Supplementary Table 1). Additionally, even more studies (67.44%, 58 out of 86) demonstrated that the normal participants consistently preferred the disadvantageous deck B. These findings significantly challenge the basic assumption of the IGT and suggest that evidence of PDB phenomenon is obscured by the use of the measure (C + D) − (A + B). We therefore recommend that future studies should investigate and compare the individual deck selections of clinical participants based on the consistent performance of the control participants.

### A New Raising Perspective: Gain–Loss Frequency

The preference for bad deck B shown by normal participants in the IGT was first demonstrated by Wilder et al. (1998), and the phenomenon has since been documented by other researchers (Toplak et al., 2005; Fernie and Tunney, 2006; Lin et al., 2007, 2013; Takano et al., 2010; Steingroever et al., 2013). Prior studies have defined participants’ preferences for bad deck B and good deck D in the IGT as the “gain-loss frequency effect” (Lin et al., 2007; Chiu et al., 2008), as the preference is associated with the high-frequency gain structure (i.e., nine gains, one loss) of both bad deck B and good deck D. The observed preference also implies that, under uncertainty conditions, control participants will use a “gain-stay loss-randomize” strategy, meaning that the probability of choosing the same deck will increase when participants face continuous gains, whereas the choice will be randomized when they face loss (Chiu et al., 2008; Worthy et al., 2013b; Lin et al., 2016). This strategy has been employed in recent IGT-related model studies (Worthy et al., 2013b; Lin et al., 2016).

Notably, the findings of our research depart from the original IGT study by Bechara et al. (1994)who proposed that normal (control group) participants would form a “somatic marker” (Damasio, 1994) when experiencing the gains and losses in the IGT and gradually develop a sensitivity to the long-term outcome—that is, preferring advantageous decks C and D and avoiding disadvantageous decks A and B. However, other studies (Wilder et al., 1998; Toplak et al., 2005; Fernie and Tunney, 2006; Lin et al., 2007, 2013; Takano et al., 2010; Steingroever et al., 2013) and the current findings have failed to replicate their results obtained in relation to normal participants. The present study also supports the argument that the PDB phenomenon should be evaluated in contemporary IGT-related studies given the apparent inconsistency with respect to the original IGT hypothesis (Chiu et al., 2018). In other words, the hypothesis proposed in the original IGT study should be carefully reconsidered and revised.

## Conclusion

The present review found that in over 60% of IGT studies, most normal (control group) participants favored the disadvantageous deck B and consistently applied a gain–loss frequency strategy. These findings are incongruent with the original inference made by Bechara and Damasio (2002), Ekhtiari et al. (2009), and Bakos et al. (2010)that the poor performance of normal participants was due to individual and cultural differences. The PDB phenomenon and the influence of gain–loss frequency in the IGT might be obscured by the analysis and presentation methodology being principally based on the net score measure (C + D) − (A + B). Considering the present integrative review and analysis of 958 studies, we conclude that gain–loss frequency could be a cross-cultural factor during decision making under dynamic-uncertain conditions.

## Data Availability Statement

All datasets generated for this study are included in the article/Supplementary Material.

## Author Contributions

Y-CC initiated this research topic and C-HL focused on refining the issue. Y-CC and C-HL constructed the research strategy and developed its main structure. Y-CC, C-HL, and W-KL undertook the literature review and defined the research database. Y-CC, C-HL, and W-KL developed the idea of mapping the geographical distribution of IGT-related studies for this manuscript, and W-KL created the corresponding artwork. W-KL completed the first round of the literature data collection, defined the categorization criteria and data administration, and drafted the preliminary manuscript. C-JL completed the second round of literature data collection, redefined the categorization criteria, and finalized the data re-categorization, as well as providing some interpretation. L-HL created the initial preliminary uncompleted draft in Chinese. W-KL, C-JL, C-HL, and Y-CC undertook several rounds of discussion, and all authors finalized and approved the manuscript.

## Conflict of Interest

The authors declare that the research was conducted in the absence of any commercial or financial relationships that could be construed as a potential conflict of interest.

## References

[B1] Adida M. Jollant F. Clark L. Besnier N. Guillaume S. Kaladjian A.(2011). Trait-related decision-making impairment in the three phases of bipolar disorder. *Biol. Psychiatry* 70 357–365. 10.1016/j.biopsych.2011.01.018 21429477

[B2] Ahn W. Y. Busemeyer J. R. Wagenmakers E. J. Stout J. C.(2008). Comparison of decision learning models using the generalization criterion method. *Cogn. Sci.* 32 1376–1402. 10.1080/03640210802352992 21585458

[B3] Alameda-Bailen J. R. Paíno-Quesada S. Mogedas Valladares A. I.(2012). [Decision making in cannabis users]. *Adicciones* 24 161–172. 22648319

[B4] Alameda-Bailen J. R. Salguero-Alcañiz M. P. Merchan-Clavellino A. Paíno-Quesada S.(2014). [Cognitive mechanisms in risky decision-making in cannabis users]. *Adicciones* 26 146–158. 25225731

[B5] Alarcon D. Amian J. G. Sanchez-Medina J. A.(2015). Enhancing emotion-based learning in decision-making under uncertainty. *Psicothema* 27 368–373. 10.7334/psicothema2015.45 26493575

[B6] Bakos D. S. Denburg N. Fonseca R. P. Parente M. A. D. M. P.(2010). A cultural study on decision making: performance differences on the Iowa gambling task between selected groups of Brazilians and Americans. *Psychol. Neurosci.* 3 101–107. 10.3922/j.psns.2010.1.013

[B7] Bark R. Dieckmann S. Bogerts B. Northoff G.(2005). Deficit in decision making in catatonic schizophrenia: an exploratory study. *Psychiatry Res.* 134 131–141. 10.1016/j.psychres.2004.04.013 15840414

[B8] Bechara A.(2007). *Iowa Gambling Task Professional Manual.* Lutz: Psychological Assessment Resources,Inc.

[B9] Bechara A.(2016). *Iowa Gambling Task, Version 2 Professional Manual.* Lutz: Psychological Assessment Resources, Inc.

[B10] Bechara A. Damasio A. R. Damasio H. Anderson S. W.(1994). Insensitivity to future consequences following damage to human prefrontal cortex. *Cognition* 50 7–15. 10.1016/0010-0277(94)90018-3 8039375

[B11] Bechara A. Damasio H.(2002). Decision-making and addiction (part I): impaired activation of somatic states in substance dependent individuals when pondering decisions with negative future consequences. *Neuropsychologia* 40 1675–1689. 10.1016/s0028-3932(02)00015-5 11992656

[B12] Bechara A. Damasio H. Damasio A. R. Lee G. P.(1999). Different contributions of the human amygdala and ventromedial prefrontal cortex to decision-making. *J. Neurosci.* 19 5473–5481. 10.1523/jneurosci.19-13-05473.1999 10377356 PMC6782338

[B13] Bechara A. Damasio H. Tranel D. Anderson S. W.(1998). Dissociation Of working memory from decision making within the human prefrontal cortex. *J. Neurosci.* 18 428–437. 10.1523/jneurosci.18-01-00428.1998 9412519 PMC6793407

[B14] Bechara A. Damasio H. Tranel D. Damasio A. R.(1997). Deciding advantageously before knowing the advantageous strategy. *Science* 275 1293–1295. 10.1126/science.275.5304.1293 9036851

[B15] Bechara A. Tranel D. Damasio H.(2000). Characterization of the decision-making deficit of patients with ventromedial prefrontal cortex lesions. *Brain* 123(Pt 11), 2189–2202. 10.1093/brain/123.11.2189 11050020

[B16] Beitz K. M. Salthouse T. A. Davis H. P.(2014). Performance on the Iowa gambling task: from 5 to 89 years of age. *J. Exp. Psychol. Gen.* 143 1677–1689. 10.1037/a0035823 24512562 PMC4115037

[B17] Besnard J. Allain P. Aubin G. Chauvire V. Etcharry-Bouyx F. Le Gall D.(2015). Decision-making of prefrontal patients with the Iowa gambling task: unexpected spared performances and preliminary evidence for the need of alternative measures. *Clin. Neuropsychol.* 29 509–521. 10.1080/13854046.2015.1050458 26053240

[B18] Besnard J. Le Gall D. Chauvire V. Aubin G. Etcharry-Bouyx F. Allain P.(2016). Discrepancy between social and nonsocial decision-making under uncertainty following prefrontal lobe damage: the impact of an interactionist approach. *Soc. Neurosci.* 12 430–447. 10.1080/17470919.2016.1182066 27109748

[B19] Brown E. C. Hack S. M. Gold J. M. Carpenter W. T. Jr. Fischer B. A. Prentice K. P.(2015). Integrating frequency and magnitude information in decision-making in schizophrenia: an account of patient performance on the Iowa gambling task. *J. Psychiatr. Res.* 66-67 16–23. 10.1016/j.jpsychires.2015.04.007 25959618 PMC4458199

[B20] Buelow M. T. Suhr J. A.(2014). Risky decision making in smoking and nonsmoking college students: examination of Iowa Gambling Task performance by deck type selections. *Appl. Neuropsychol. Child* 3 38–44. 10.1080/21622965.2012.691065 24236940

[B21] Bull P. N. Tippett L. J. Addis D. R.(2015). Decision making in healthy participants on the Iowa gambling task: new insights from an operant approach. *Front. Psychol.* 6:391. 10.3389/fpsyg.2015.00391 25904884 PMC4387474

[B22] Cardoso C. D. O. Branco L. D. Cotrena C. Kristensen C. H. Schneider Bakos D. D. Fonseca R. P.(2014). The impact of frontal and cerebellar lesions on decision making: evidence from the Iowa gambling task. *Front. Neurosci.* 8:61. 10.3389/fnins.2014.00061 24782697 PMC3986592

[B23] Caroselli J. S. Hiscock M. Scheibel R. S. Ingram F.(2006). The simulated gambling paradigm applied to young adults: an examination of university students’ performance. *Appl. Neuropsychol.* 13 203–212. 10.1207/s15324826an1304_1 17362140

[B24] Carvalho J. C. Cardoso Cde O. Shneider-Bakos D. Kristensen C. H. Fonseca R. P.(2012). The effect of age on decision making according to the Iowa gambling task. *Span. J. Psychol.* 15 480–486. 10.5209/rev_sjop.2012.v15.n2.38858 22774421

[B25] Chiu Y. C. Huang J. T. Duann J. R. Lin C. H.(2018). Editorial: twenty years after the iowa gambling task: rationality, emotion, and decision-making. *Front. Psychol.* 8:2353. 10.3389/fpsyg.2017.02353 29422876 PMC5788943

[B26] Chiu Y. C. Lin C. H.(2007). Is deck C an advantageous deck in the Iowa gambling task? *Behav. Brain Funct.* 3:37. 10.1186/1744-9081-3-37 17683599 PMC1995208

[B27] Chiu Y. C. Lin C. H. Huang J. T.(2012). “Prominent deck B phenomenon: are decision-makers sensitive to long-term outcome in the Iowa gambling task?,” in *Psychology of Gambling: New Research*, ed. Cavanna A.(New York, NY: Nova), 93–118.

[B28] Chiu Y. C. Lin C. H. Huang J. T. Lin S. Lee P. L. Hsieh J. C.(2008). Immediate gain is long-term loss: are there foresighted decision makers in the Iowa gambling task? *Behav. Brain Funct.* 4:13. 10.1186/1744-9081-4-13 18353176 PMC2324107

[B29] Cotrena C. Branco L. D. Zimmermann N. Cardoso C. O. Grassi-Oliveira R. Fonseca R. P.(2014). Impaired decision-making after traumatic brain injury: the Iowa gambling task. *Brain Inj.* 28 1070–1075. 10.3109/02699052.2014.896943 24654680

[B30] Damasio A. R.(1994). *Descartes’ Error: Emotion, Reason, and the Human Brain.* New York, NY: G. P. Putnam’s Sons.

[B31] Denburg N. L. Tranel D. Bechara A.(2005). The ability to decide advantageously declines prematurely in some normal older persons. *Neuropsychologia* 43 1099–1106. 10.1016/j.neuropsychologia.2004.09.012 15769495

[B32] Dunn B. D. Dalgleish T. Lawrence A. D.(2006). The somatic marker hypothesis: a critical evaluation. *Neurosci. Biobehav. Rev.* 30 239–271. 10.1016/j.neubiorev.2005.07.001 16197997

[B33] Ekhtiari H. Behzadi A. Dehghani M. Jannati A. Mokri A.(2009). Prefer a cash slap in your face over credit for halva. *Judgm. Decis. Mak.* 4 534–542.

[B34] Escartin G. Junque C. Juncadella M. Gabarros A. de Miquel M. A. Rubio F.(2012). Decision-making impairment on the Iowa Gambling Task after endovascular coiling or neurosurgical clipping for ruptured anterior communicating artery aneurysm. *Neuropsychology* 26 172–180. 10.1037/a0024336 22251310

[B35] Evans K. L. Hampson E.(2015). Sex differences on prefrontally-dependent cognitive tasks. *Brain Cogn.* 93 42–53. 10.1016/j.bandc.2014.11.006 25528435

[B36] Fernie G. Tunney R. J.(2006). Some decks are better than others: the effect of reinforcer type and task instructions on learning in the Iowa gambling task. *Brain Cogn.* 60 94–102. 10.1016/j.bandc.2005.09.011 16271818

[B37] Fum D. Napoli A. Stocco A.(2008). “Somatic markers and frequency effects: does emotion really play a role on decision making in the iowa gambling task?,” in *Proceedings of the 30th Annual Conference of the Cognitive Science Society*, Washington, DC.

[B38] Gansler D. A. Jerram M. W. Vannorsdall T. D. Schretlen D. J.(2011). Comparing alternative metrics to assess performance on the Iowa gambling task. *J. Clin. Exp. Neuropsychol.* 33 1040–1048. 10.1080/13803395.2011.596820 21916658

[B39] Gansler D. A. Jerram M. W. Vannorsdall T. D. Schretlen D. J.(2012). Hierarchical organization of cortical morphology of decision-making when deconstructing Iowa gambling task performance in healthy adults. *J. Int. Neuropsychol. Soc.* 18 585–594. 10.1017/S1355617712000215 22394607

[B40] Gescheidt T. Czekoova K. Urbanek T. Marecek R. Mikl M. Kubikova R.(2012). Iowa Gambling Task in patients with early-onset Parkinson’s disease: strategy analysis. *Neurol. Sci.* 33 1329–1335. 10.1007/s10072-012-1086-x 22526761

[B41] Hawthorne M. J. Pierce B. H.(2015). Disadvantageous deck selection in the iowa gambling task: the effect of cognitive load. *Eur. J. Psychol.* 11 335–348. 10.5964/ejop.v11i2.931 27247661 PMC4873115

[B42] Hong X. Zheng L. Li X.(2015). Impaired decision making is associated with poor inhibition control in nonpathological lottery gamblers. *J. Gambl. Stud.* 31 1617–1632. 10.1007/s10899-014-9509-7 25348253

[B43] Hori H. Yoshimura R. Katsuki A. Atake K. Nakamura J.(2014). Relationships between brain-derived neurotrophic factor, clinical symptoms, and decision-making in chronic schizophrenia: data from the Iowa gambling task. *Front. Behav. Neurosci.* 8:417. 10.3389/fnbeh.2014.00417 25538582 PMC4255599

[B44] Horstmann A. Villringer A. Neumann J.(2012). Iowa gambling task: there is more to consider than long-term outcome. Using a linear equation model to disentangle the impact of outcome and frequency of gains and losses. *Front. Neurosci.* 6:61. 10.3389/fnins.2012.00061 22593730 PMC3350871

[B45] Huang Y. H. Wood S. Berger D. E. Hanoch Y.(2015). Age differences in experiential and deliberative processes in unambiguous and ambiguous decision making. *Psychol. Aging* 30 675–687. 10.1037/pag0000038 26280384

[B46] Jollans L. Whelan R. Venables L. Turnbull O. H. Cella M. Dymond S.(2017). Computational EEG modelling of decision making under ambiguity reveals spatio-temporal dynamics of outcome evaluation. *Behav. Brain Res.* 321 28–35. 10.1016/j.bbr.2016.12.033 28034803

[B47] Kester H. M. Sevy S. Yechiam E. Burdick K. E. Cervellione K. L. Kumra S.(2006). Decision-making impairments in adolescents with early-onset schizophrenia. *Schizophr. Res.* 85 113–123. 10.1016/j.schres.2006.02.028 16733084

[B48] Kim Y. T. Lee K. U. Lee S. J.(2009). Deficit in decision-making in chronic, stable schizophrenia: from a reward and punishment perspective. *Psychiatry Investig.* 6 26–33. 10.4306/pi.2009.6.1.26 20046370 PMC2796041

[B49] Kim Y. T. Sohn H. Jeong J.(2011). Delayed transition from ambiguous to risky decision making in alcohol dependence during Iowa Gambling Task. *Psychiatry Res.* 190 297–303. 10.1016/j.psychres.2011.05.003 21676471

[B50] Kim Y. T. Sohn H. Kim S. Oh J. Peterson B. S. Jeong J.(2012). Disturbances of motivational balance in chronic schizophrenia during decision-making tasks. *Psychiatry Clin. Neurosci.* 66 573–581. 10.1111/j.1440-1819.2012.02403.x 23252923

[B51] Kloeters S. Bertoux M. O’Callaghan C. Hodges J. R. Hornberger M.(2013). Money for nothing - Atrophy correlates of gambling decision making in behavioural variant frontotemporal dementia and Alzheimer’s disease. *Neuroimage Clin.* 2 263–272. 10.1016/j.nicl.2013.01.011 24179781 PMC3778267

[B52] Lavin C. San Martin R. Rosales Jubal E.(2014). Pupil dilation signals uncertainty and surprise in a learning gambling task. *Front. Behav. Neurosci.* 7:218. 10.3389/fnbeh.2013.00218 24427126 PMC3879532

[B53] Le Berre A. P. Rauchs G. La Joie R. Mezenge F. Boudehent C. Vabret F.(2014). Impaired decision-making and brain shrinkage in alcoholism. *Eur. Psychiatry* 29 125–133. 10.1016/j.eurpsy.2012.10.002 23182846

[B54] Lee W. K. Su Y. A. Song T. J. Chiu Y. C. Lin C. H.(2014). Are normal decision-makers sensitive to changes in value contrast under uncertainty? Evidence from the Iowa gambling task. *PLoS One* 9:e101878. 10.1371/journal.pone.0101878 25036094 PMC4103768

[B55] Lee Y. Kim Y. T. Seo E. Park O. Jeong S. H. Kim S. H.(2007). Dissociation of emotional decision-making from cognitive decision-making in chronic schizophrenia. *Psychiatry Res.* 152 113–120. 10.1016/j.psychres.2006.02.001 17462743

[B56] LeGris J. Toplak M. Links P. S.(2014). Affective decision making in women with borderline personality disorder. *J. Pers. Disord.* 28 698–719. 10.1521/pedi_2014_28_140 24845226

[B57] Lin C. H. Chiu Y. C. Lee P. L. Hsieh J. C.(2007). Is deck B a disadvantageous deck in the Iowa gambling task? *Behav. Brain Funct.* 3:16. 10.1186/1744-9081-3-16 17362508 PMC1839101

[B58] Lin C. H. Lin Y. K. Song T. J. Huang J. T. Chiu Y. C.(2016). A simplified model of choice behavior under uncertainty. *Front. Psychol.* 7:1201. 10.3389/fpsyg.2016.01201 27582715 PMC4987346

[B59] Lin C. H. Song T. J. Chen Y. Y. Lee W. K. Chiu Y. C.(2013). Reexamining the validity and reliability of the clinical version of the iowa gambling task: evidence from a normal subject group. *Front. Psychol.* 4:220. 10.3389/fpsyg.2013.00220 23755026 PMC3665927

[B60] Ma S. Zang Y. Cheung V. Chan C. C.(2015). Importance of punishment frequency in the Iowa gambling task: an fMRI study. *Brain Imaging Behav.* 9 899–909. 10.1007/s11682-015-9353-0 25724688

[B61] Martino D. J. Bucay D. Butman J. T. Allegri R. F.(2007). Neuropsychological frontal impairments and negative symptoms in schizophrenia. *Psychiatry Res.* 152 121–128. 10.1016/j.psychres.2006.03.002 17507100

[B62] Martino D. J. Strejilevich S. A. Torralva T. Manes F.(2011). Decision making in euthymic bipolar I and bipolar II disorders. *Psychol. Med.* 41 1319–1327. 10.1017/S0033291710001832 20860871

[B63] Matsuzawa D. Shirayama Y. Niitsu T. Hashimoto K. Iyo M.(2015). Deficits in emotion based decision-making in schizophrenia; a new insight based on the Iowa gambling task. *Prog. Neuropsychopharmacol. Biol. Psychiatry* 57 52–59. 10.1016/j.pnpbp.2014.10.007 25455588

[B64] Miller M. Sheridan M. Cardoos S. L. Hinshaw S. P.(2013). Impaired decision-making as a young adult outcome of girls diagnosed with attention-deficit/hyperactivity disorder in childhood. *J. Int. Neuropsychol. Soc.* 19 110–114. 10.1017/S1355617712000975 23089192 PMC3959801

[B65] Mogedas Valladares A. I. Alameda-Bailen J. R.(2011). [Decision-making in drug-dependent patients]. *Adicciones* 23 277–287. 22249893

[B66] North N. T. O’Carroll R. E.(2001). Decision making in patients with spinal cord damage: afferent feedback and the somatic marker hypothesis. *Neuropsychologia* 39 521–524. 10.1016/s0028-3932(00)00107-x 11254934

[B67] Northoff G. Grimm S. Boeker H. Schmidt C. Bermpohl F. Heinzel A.(2006). Affective judgment and beneficial decision making: ventromedial prefrontal activity correlates with performance in the Iowa gambling task. *Hum. Brain Mapp.* 27 572–587. 10.1002/hbm.20202 16372256 PMC6871437

[B68] O’Carroll R. E. Papps B. P.(2003). Decision making in humans: the effect of manipulating the central noradrenergic system. *J. Neurol. Neurosurg. Psychiatry* 74 376–378. 10.1136/jnnp.74.3.376 12588933 PMC1738336

[B69] Okdie B. M. Buelow M. T. Bevelhymer-Rangel K.(2016). It’s all in how you think about it: construal level and the Iowa gambling task. *Front. Neurosci.* 10:2. 10.3389/fnins.2016.00002 26834531 PMC4722111

[B70] Overman W. H.(2004). Sex differences in early childhood, adolescence, and adulthood on cognitive tasks that rely on orbital prefrontal cortex. *Brain Cogn.* 55 134–147. 10.1016/S0278-2626(03)00279-3 15134848

[B71] Pedersen A. Goder R. Tomczyk S. Ohrmann P.(2017). Risky decision-making under risk in schizophrenia: a deliberate choice? *J. Behav. Ther. Exp. Psychiatry* 56 57–64. 10.1016/j.jbtep.2016.08.004 27568887

[B72] Penolazzi B. Leone L. Russo P. M.(2013). Individual differences and decision making: when the lure effect of gain is a matter of size. *PLoS One* 8:e58946. 10.1371/journal.pone.0058946 23484058 PMC3590131

[B73] Petry N. M.(2001). Substance abuse, pathological gambling, and impulsiveness. *Drug Alcohol Depend.* 63 29–38. 10.1016/s0376-8716(00)00188-5 11297829

[B74] Petry N. M. Bickel W. K. Arnett M.(1998). Shortened time horizons and insensitivity to future consequences in heroin addicts. *Addiction* 93 729–738. 10.1046/j.1360-0443.1998.9357298.x 9692271

[B75] Piper B. Mueller S. T. Talebzadeh S. Ki M. J.(2016). Evaluation of the validity of the psychology experiment building language tests of vigilance, auditory memory, and decision making. *PeerJ* 4:e1772. 10.7717/peerj.1772 27014512 PMC4806597

[B76] Reavis R. Overman W. H.(2001). Adult sex differences on a decision-making task previously shown to depend on the orbital prefrontal cortex. *Behav. Neurosci.* 115, 196–206. 10.1037/0735-7044.115.1.196 11256443

[B77] Ritter L. M. Meador-Woodruff J. H. Dalack G. W.(2004). Neurocognitive measures of prefrontal cortical dysfunction in schizophrenia. *Schizophr. Res.* 68 65–73. 10.1016/S0920-9964(03)00086-0 15037340

[B78] Rodriguez-Sanchez J. M. Crespo-Facorro B. Perez-Iglesias R. Gonzalez-Blanch C. Alvarez-Jimenez M. Llorca J.(2005). Prefrontal cognitive functions in stabilized first-episode patients with schizophrenia spectrum disorders: a dissociation between dorsolateral and orbitofrontal functioning. *Schizophr. Res.* 77 279–288. 10.1016/j.schres.2005.04.023 15950437

[B79] Seeley C. J. Beninger R. J. Smith C. T.(2014). Post learning sleep improves cognitive-emotional decision-making: evidence for a ‘deck B sleep effect’ in the Iowa Gambling Task. *PLoS One* 9:e112056. 10.1371/journal.pone.0112056 25409323 PMC4237319

[B80] Seeley C. J. Smith C. T. MacDonald K. J. Beninger R. J.(2016). Ventromedial prefrontal theta activity during rapid eye movement sleep is associated with improved decision-making on the Iowa gambling task. *Behav. Neurosci.* 130 271–280. 10.1037/bne0000123 26820586

[B81] Sevy S. Burdick K. E. Visweswaraiah H. Abdelmessih S. Lukin M. Yechiam E.(2007). Iowa gambling task in schizophrenia: a review and new data in patients with schizophrenia and co-occurring cannabis use disorders. *Schizophr. Res.* 92 74–84. 10.1016/j.schres.2007.01.005 17379482 PMC2039912

[B82] Shurman B. Horan W. P. Nuechterlein K. H.(2005). Schizophrenia patients demonstrate a distinctive pattern of decision-making impairment on the Iowa gambling task. *Schizophr. Res.* 72 215–224. 10.1016/j.schres.2004.03.020 15560966

[B83] Smart C. M. Krawitz A.(2015). The impact of subjective cognitive decline on Iowa Gambling Task performance. *Neuropsychology* 29 971–987. 10.1037/neu0000204 26011116

[B84] Steingroever H. Wetzels R. Horstmann A. Neumann J. Wagenmakers E. J.(2013). Performance of healthy participants on the Iowa gambling task. *Psychol. Assess.* 25 180–193. 10.1037/a0029929 22984804

[B85] Takano Y. Takahashi N. Tanaka D. Hironaka N.(2010). Big losses lead to irrational decision-making in gambling situations: relationship between deliberation and impulsivity. *PLoS One* 5:e9368. 10.1371/journal.pone.0009368 20186323 PMC2826400

[B86] Tchanturia K. Liao P. C. Forcano L. Fernandez-Aranda F. Uher R. Treasure J.(2012). Poor decision making in male patients with anorexia nervosa. *Eur. Eat Disord. Rev.* 20 169–173. 10.1002/erv.1154 21830260

[B87] Tomb I. Hauser M. Deldin P. Caramazza A.(2002). Do somatic markers mediate decisions on the gambling task? *Nat. Neurosci.* 5 1103–1104. 10.1038/nn1102-1103 12403997

[B88] Toplak M. E. Jain U. Tannock R.(2005). Executive and motivational processes in adolescents with Attention-Deficit-Hyperactivity Disorder (ADHD). *Behav. Brain Funct.* 1:8. 10.1186/1744-9081-1-8 15982413 PMC1183187

[B89] Upton D. J. Kerestes R. Stout J. C.(2012). Comparing the Iowa and soochow gambling tasks in opiate users. *Front. Neurosci.* 6:34. 10.3389/fnins.2012.00034 22435045 PMC3303110

[B90] van den Bos R. Harteveld M. Stoop H.(2009). Stress and decision-making in humans: performance is related to cortisol reactivity, albeit differently in men and women. *Psychoneuroendocrinology* 34 1449–1458. 10.1016/j.psyneuen.2009.04.016 19497677

[B91] van den Bos R. Homberg J. de Visser L.(2013). A critical review of sex differences in decision-making tasks: focus on the Iowa gambling task. *Behav. Brain Res.* 238 95–108. 10.1016/j.bbr.2012.10.002 23078950

[B92] van Toor D. Roozen H. G. Evans B. E. Rombout L. Van de Wetering B. J. Vingerhoets A. J.(2011). The effects of psychiatric distress, inhibition, and impulsivity on decision making in patients with substance use disorders: a matched control study. *J. Clin. Exp. Neuropsychol.* 33 161–168. 10.1080/13803395.2010.493300 20628947

[B93] Vassileva J. Ahn W. Y. Weber K. M. Busemeyer J. R. Stout J. C. Gonzalez R.(2013). Computational modeling reveals distinct effects of HIV and history of drug use on decision-making processes in women. *PLoS One* 8:e68962. 10.1371/journal.pone.0068962 23950880 PMC3737214

[B94] Visagan R. Xiang A. Lamar M.(2012). Comparison of deck- and trial-based approaches to advantageous decision making on the Iowa gambling task. *Psychol. Assess.* 24 455–463. 10.1037/a0025932 22040516

[B95] Visser-Keizer A. C. Westerhof-Evers H. J. Gerritsen M. J. van der Naalt J. Spikman J. M.(2016). To fear is to gain? The role of fear recognition in risky decision making in TBI patients and healthy controls. *PLoS One* 11:e0166995. 10.1371/journal.pone.0166995 27870900 PMC5117759

[B96] Viswanath B. Janardhan Reddy Y. C. Kumar K. J. Kandavel T. Chandrashekar C. R.(2009). Cognitive endophenotypes in OCD: a study of unaffected siblings of probands with familial OCD. *Prog. Neuropsychopharmacol. Biol. Psychiatry* 33 610–615. 10.1016/j.pnpbp.2009.02.018 19272409

[B97] Wilder K. E. Weinberger D. R. Goldberg T. E.(1998). Operant conditioning and the orbitofrontal cortex in schizophrenic patients: unexpected evidence for intact functioning. *Schizophr. Res.* 30 169–174. 10.1016/s0920-9964(97)00135-7 9549781

[B98] Wolk J. Sutterlin S. Koch S. Vogele C. Schulz S. M.(2014). Enhanced cardiac perception predicts impaired performance in the Iowa Gambling Task in patients with panic disorder. *Brain Behav.* 4 238–246. 10.1002/brb3.206 24683516 PMC3967539

[B99] Worthy D. A. Hawthorne M. J. Otto A. R.(2013a). Heterogeneity of strategy use in the Iowa gambling task: a comparison of win-stay/lose-shift and reinforcement learning models. *Psychon. Bull. Rev.* 20 364–371. 10.3758/s13423-012-0324-9 23065763

[B100] Worthy D. A. Pang B. Byrne K. A.(2013b). Decomposing the roles of perseveration and expected value representation in models of the Iowa gambling task. *Front. Psychol.* 4:640. 10.3389/fpsyg.2013.00640 24137137 PMC3786232

[B101] Wright R. J. Rakow T. Russo R.(2017). Go for broke: the role of somatic states when asked to lose in the Iowa gambling task. *Biol. Psychol.* 123 286–293. 10.1016/j.biopsycho.2016.10.014 27984085

[B102] Yechiam E. Telpaz A. Krupenia S. Rafaeli A.(2016). Unhappiness intensifies the avoidance of frequent losses while happiness overcomes it. *Front. Psychol.* 7:1703. 10.3389/fpsyg.2016.01703 27853443 PMC5089969

[B103] Zamarian L. Sinz H. Bonatti E. Gamboz N. Delazer M.(2008). Normal aging affects decisions under ambiguity, but not decisions under risk. *Neuropsychology* 22 645–657. 10.1037/0894-4105.22.5.645 18763884

[B104] Zhang L. Dong Y. Ji Y. Tao R. Chen X. Ye J.(2015a). Trait-related decision making impairment in obsessive-compulsive disorder: evidence from decision making under ambiguity but not decision making under risk. *Sci. Rep.* 5:17312. 10.1038/srep17312 26601899 PMC4658550

[B105] Zhang L. Tang J. Dong Y. Ji Y. Tao R. Liang Z.(2015b). Similarities and differences in decision-making impairments between autism spectrum disorder and schizophrenia. *Front. Behav. Neurosci.* 9:259. 10.3389/fnbeh.2015.00259 26441583 PMC4585296

[B106] Zhang L. Wang K. Zhu C. Yu F. Chen X.(2015c). Trait anxiety has effect on decision making under ambiguity but not decision making under risk. *PLoS One* 10:e0127189. 10.1371/journal.pone.0127189 26000629 PMC4441420

