# Supplementary material for: Recollecting Cross-Cultural Evidences: Are Decision Makers Really Foresighted in Iowa Gambling Task?
Source: Front Psychol. 2020 Dec 21;11:537219. doi: 10.3389/fpsyg.2020.537219 (PMC7779794; doi:10.3389/fpsyg.2020.537219)
Supplement: Supplementary file 1 [file Table_1.docx]

Supplementary table 1. Net score (C+D) - (A+B) of normal participants in 86 IGT-related studies which presented individual deck selections

| Authors | Source of Study |  |  |  |
| --- | --- | --- | --- | --- |
|  |  | **Net score (C+D) – (A+B)** | **Is the net score (C+D) – (A+B) < 10** | **Note** |
| Petry et al. (1998) | US | 17.70 | No | ≈ |
| Petry (2001) | US | 20.20 | No | ≈ |
| North and O'Carroll (2001) | GB | 41.80 | No | ≈ |
| O'Carroll and Papps (2003) | GB | 14.70 | No | ≈ |
| Overman (2004) | US | 15.50 | No | ≈ |
|  |  | 14.65 |  |  |
|  |  | 34.15 |  |  |
|  |  | 21.10 |  |  |
| Shurman et al. (2005) | US | 31.60 | No |  |
| Bark et al. (2005) | DE | -3.10 | Yes | ≈ |
| Rodriguez-Sanchez et al. (2005) | ES | 1.90 | Yes |  |
| Fernie and Tunney (2006) | GB | -0.80 | Yes | ≈ |
|  |  | -6.20 |  |  |
| Northoff et al. (2006) | DE | 15.82 | No | ≈ |
| Kester et al. (2006) | US | 8.00 | Yes |  |
| Sevy et al. (2007) | US | 1.20 | Yes |  |
| Lee et al. (2007) | KR | 17.40 | No |  |
| Martino et al. (2007) | AR | 17.06 | No |  |
| Zamarian et al. (2008) | AT | 32.10 | No | ≈ |
|  |  | 16.40 |  |  |
| Ahn et al. (2008) | US | 24.84 | No | ≈ |
| Viswanath et al. (2009) | IN | 13.76 | No |  |
| **van den Bos et al. (2009)** | **NL** | **18.40** | **Yes** | **≈** |
|  |  | **7.00** |  |  |
| Kim et al. (2009) | KR | 14.00 | No |  |
| van Toor et al. (2011) | NL | 21.03 | No |  |
| Martino et al. (2011) | AR | 16.40 | No | ≈ |
| Adida et al. (2011) | GB  FR | 16.70 | No | ≈ |
| Kim et al. (2011) | KR | 23.52 | No |  |
| Tchanturia et al. (2012) | GB ES | 14.68 | No |  |
|  |  | 20.80 |  |  |
| Gansler et al. (2011) | US | 11.58 | No |  |
| Visagan et al. (2012) | GB | 16.80 | No |  |
| Mogedas Valladares and Alameda-Bailen (2011) | ES | 17.15 | No |  |
| Escartin et al. (2012) | ES | 11.10 | No |  |
| Gansler et al. (2012) | US | 9.77 | Yes |  |
| Upton et al. (2012) | AU | -2.50 | Yes | ≈ |
| Gescheidt et al. (2012) | CZ | 10.30 | No |  |
| Horstmann et al. (2012) | DE | 4.99 | Yes | ≈ |
| Alameda-Bailen et al. (2012) | ES | 19.12 | No |  |
| Carvalho et al. (2012) | BR | 9.50 | Yes |  |
|  |  | 3.93 |  |  |
| Steingroever et al. (2013) | NL | 0.00 | Yes | ≈ |
| Worthy et al. (2013a) | US | 2.06 | Yes | ≈ |
| **van den Bos et al. (2013)** | **NL** | **5.70** | **Yes** |  |
|  |  | **12.30** |  |  |
| Miller et al. (2013) | US | 8.11 | Yes | ≈ |
| Le Berre et al. (2014) | FR | 18.60 | No |  |
| Kim et al. (2012) | KR | 16.90 | No |  |
| Penolazzi et al. (2013) | IT | 10.12 | No |  |
| Lin et al. (2013) | TW | 1.56 | Yes |  |
| Vassileva et al. (2013) | US | 0.17 | Yes |  |
| Kloeters et al. (2013) | AU | 18.90 | No |  |
| Buelow and Suhr (2014) | US | 5.25 | Yes | ≈ |
| Lavin et al. (2014) | CL | -12.20 | Yes | ≈ |
| Beitz et al. (2014) | US | 20.70 | No | ≈ |
|  |  | 12.30 |  |  |
| Cotrena et al. (2014) | BR | 16.70 | No | ≈ |
| Wolk et al. (2014) | DE | -3.38 | Yes | ≈ |
| Cardoso et al. (2014) | BR | 23.00 | No |  |
| LeGris et al. (2014) | CA | 21.07 | No |  |
| Lee et al. (2014) | TW | 5.48 | Yes | ≈ |
| Alameda-Bailen et al. (2014) | ES | 22.10 | No |  |
| Hong et al. (2015) | CN | 19.10 | No | ≈ |
| Seeley et al. (2014) | CA | -1.10 | Yes | ≈ |
| Matsuzawa et al. (2015) | JP | 0.50 | Yes | ≈ |
| Evans and Hampson (2015) | CA | 9.30 | Yes | ≈ |
|  |  | -1.40 |  |  |
| Hori et al. (2014) | JP | 12.20 | No |  |
| Ma et al. (2015) | CN | -2.33 | Yes |  |
| Bull et al. (2015) | NZ | 24.00 | No |  |
| Brown et al. (2015) | US | 11.90 | No | ≈ |
| Zhang et al. (2015c) | CN | -10.10 | Yes | ≈ |
|  |  | 3.30 |  |  |
|  |  | -5.80 |  |  |
| Smart and Krawitz (2015) | CA | 20.37 | No | ≈ |
| Besnard et al. (2015) | FR | 35.90 | No |  |
| Huang et al. (2015) | US | -0.65 | Yes |  |
|  |  | -3.48 |  |  |
| Zhang et al. (2015b) | CN | 3.10 | Yes | ≈ |
| Alarcon et al. (2015) | ES | 2.20 | Yes |  |
| Zhang et al. (2015a) | CN | 3.35 | Yes |  |
| Seeley et al. (2016) | CA | -13.19 | Yes | ≈ |
| Okdie et al. (2016) | US | 2.82 | Yes | ≈ |
|  |  | 0.68 |  |  |
| Piper et al. (2016) | US | 1.50 | Yes | ≈ |
|  |  | 0.70 |  |  |
| Besnard et al. (2016) | FR | 25.40 | No |  |
| Hawthorne and Pierce (2015) | US | 14.90 | No | ≈ |
| Pedersen et al. (2017) | DE | 0.368 | Yes |  |
| Lin et al. (2016) | TW | 0.28 | Yes | ≈ |
| Yechiam et al. (2016) | IL | 21.90 | No | ≈ |
| Visser-Keizer et al. (2016) | NL | 11.60 | No | ≈ |
| Wright et al. (2017) | GB | 15.20 | No | ≈ |
| Jollans et al. (2017) | GB | -6.25 | Yes | ≈ |
| Bechara et al. (1994) | US | 40.00 | No | ≈ |
| Wilder et al. (1998) | US | 6.00 | Yes | ≈ |
| Tomb et al. (2002) | US | 32.00 | No | ≈ |
| Ritter et al. (2004) | US | 14.40 | No |  |
| Caroselli et al. (2006) | US | -14.00 | Yes | ≈ |
| Fum et al. (2008) | IT | 4.57 | Yes | ≈ |
| Chiu et al. (2012) | TW | 0.75 | Yes | ≈ |

Note: The two studies that marked bold in the Supplementary Table 1 showed only one of two experimental conditions existed the performance of (C+D) - (A+B) < 10.

≈: Net score (C+D) - (A+B) data were calculated according to individual deck data shown in Table 1

Note without special signs above: Net score (C+D) - (A+B) data were obtained from original studies
